# Supplementary material for: Clinical Characteristics and Outcomes in 314 Japanese Patients with Bacterial Endophthalmitis: A Multicenter Cohort Study from J-CREST
Source: Pathogens. 2021 Mar 24;10(4):390. doi: 10.3390/pathogens10040390 (PMC8063932; doi:10.3390/pathogens10040390)
Supplement: Supplementary file 1 [file pathogens-10-00390-s001.zip › pathogens-1151745 supplementary- proof/Table S2-1.pdf]

Age	Gender M	RF=0, LF=	Type of ey	自覚症状	感因性子	主訴·眼	主訴·手	Pre-oper	Pre-oper	前房水	前房水	Classific	眼内	Post-oper	Post-oper	Post-oper	Post-oper	治療	最終的	Primary os
77	0	1	0	1	3650	1	1		2.3	0			0	0.2	0.69897	0.8	0.09691	2	1	1
32	1	1	1	14		0	0	0.6	0.22185				Candida :	0.9	0.04576	1.2	-0.0792	0	1	1
56	1	0	1	2		0	0	0.9	0.04576				Candida :	1.2	-0.0792	1.2	-0.0792	0	1	1
56	1	1	1	2		0	0	1	0				Candida :	1.5	-0.1761	1.2	-0.0792	0	1	1
57	0	0	1	14		0	0		2.6	1	Candida :	8	0		2.9		2.9	2	0	0
36	1	1	1	2		0	0	1	0				Candida :	1.2	-0.0792	1.2	-0.0792	0	1	1
69	0	0	1	7		0	0	1.2	-0.0792				Candida :	1.2	-0.0792	1	0	0	1	1
69	0	1	1	7		0	0	1	0				Candida :	1	0	0.8	0.09691	0	1	1
76	0	1	1	1		0	0		2.6	1	Streptoco	3	0		2	0.01	2	2	1	0
43	0	0	1			0	0	0.7	0.1549				Candida :	0.7	0.1549	0.7	0.1549	0	1	1
43	0	1	1			0	0	0.7	0.1549				Candida :	0.7	0.1549	0.7	0.1549	0	1	1
44	1	0	1	28		0	0	1.2	-0.0792				Candida :	1.2	-0.0792	1.2	-0.0792	0	1	1
62	0	0	0	8	2190	1	1		2.6	0			0	0.2	0.69897	0.2	0.69897	2	1	1
70	0	1	0	1	3	1	1		2.3	1	Staphyloc	2	0	0.2	0.69897	0.2	0.69897	2	1	1
48	1	1	1	1		1	1		2.6	1	Streptoco	3	1		2.3		2.3	2	0	0
76	0	1	0	1	5	0	1		2.3	1	Staphyloc	1	0	0.3	0.52288	0.3	0.52288	2	1	1
47	0	1	0	3	3	1	1		2.3	1	Staphyloc	2	0	0.1	1	0.3	0.52288	2	1	1
44	1	1	1	28		0	0	0.2	0.69897				Candida :	1	0	1	0	0	1	1
82	0	0	1	1		0	0	0.02	1.69897	0			Streptoco	0.8	0.09691	1	0	2	1	1
82	0	1	1	1		0	0	0.02	1.69897				Streptoco	0.8	0.09691	1	0	2	1	1
9	0	1	0	1	1	1	1		2.3	1	Pantoea :	7	0		2	0.3	0.52288	2	1	1
60	0	0	1			0	0	0.6	0.22185				Candida :	0.6	0.22185	0.6	0.22185	0	1	1
60	0	1	1			0	0	1	0				Candida :	1	0	1	0	0	1	1
74	1	0	1			0	0	0.8	0.09691				Candida :	1	0	1	0	0	1	1
74	1	1	1			0	0	0.8	0.09691				Candida :	1	0	1	0	0	1	1
51	0	0	1			0	0	1.2	-0.0792				Candida :	1.2	-0.0792	1.2	-0.0792	0	1	1
51	0	1	1			0	0	1.2	-0.0792				Candida :	1.2	-0.0792	1.2	-0.0792	0	1	1
40	1	0	1			0	0	1.2	-0.0792				Candida :	1.2	-0.0792	1.2	-0.0792	0	1	1
40	1	1	1			0	0	1.2	-0.0792				Candida :	1.2	-0.0792	1.2	-0.0792	0	1	1
58	0	1	0	20	21	0	1	0.3	0.52288	0				1.2	-0.0792	1.2	-0.0792	2	1	1
73	0	1	0	4	56	0	1	1.2	-0.0792	0				1.2	-0.0792	1.2	-0.0792	2	1	1
81	1	1	0	2	21	0	1	0.2	0.69897	0				0.9	0.04576	1	0	2	1	1
58	0	0	1			0	0	1	0				Candida :	1	0	1	0	0	1	1
55	1	0	1			0	0	0.07	1.1549				Candida :	0.15	0.82391	0.3	0.52288	2	1	1
64	0	0	1			0	0	1.2	-0.0792				Candida :	1.2	-0.0792	1.2	-0.0792	0	1	1
55	1	0	1			0	0	1	0				Candida :	1	0	1	0	0	1	1
55	1	1	1			0	0	1.2	-0.0792				Candida :	1	0	1	0	0	1	1
45	0	1	1	3		1	1		2.3	1	Klebsiell:	5	1	0.4	0.39794	0.6	0.22185	2	1	1
78	1	1	0	2	8	1	1		2.3	1	Staphyloc	1	0	0.2	0.69897	0.3	0.52288	2	1	1
10	1	0	0	1	1	1	1	0.6	0.22185	1	Pasteurel	7	0	0.7	0.1549	0.8	0.09691	2	1	1
67	1	0	1	12		1	1		2.6	1	Streptoco	3	0		2.9		2.9	2	0	0
63	0	1	0	1	2	1	1		2.3	1	Staphyloc	1	0	0.2	0.69897	0.3	0.52288	2	1	1
45	1	0	1	20		0	0	1.2	-0.0792				Candida :	1.2	-0.0792	1.2	-0.0792	0	1	1
88	1	0	1	10		0	0	0.02	1.69897	0				0.8	0.09691	1	0	2	1	1
88	1	1	1	2		0	0	0.6	0.22185	0				0.9	0.04576	1	0	2	1	1
63	0	0	1	6		0	0	0.1	1					0.7	0.1549	0.9	0.04576	1	1	1
81	0	1	0	60		1	1	0.08	1.09691	0				0.15	0.82391	0.15	0.82391	2		1
84	1	1	0	1	5	0	0	0.1	1	1	Staphyloc	1	0	0.9	0.04576	0.9	0.04576	2	1	1
75	1	0	0	0	4	0	0	0.1	1	0				0.02	1.69897	0.05	1.30103	2	1	0
63	0	0	0	7	18	1	1	0.1	1	0				0.5	0.30103	0.8	0.09691	2	1	1
60	0	1	1	60		0	0	0.15	0.82391	1	Candida :	8	1	0.15	0.82391	0.2	0.69897	2	1	1
60	0	0	1	1		0	0	0.04	1.39794	0				0.5	0.30103	1.2	-0.0792	2	1	1
84	0	0	0	1	6	0	0	0.6	0.22185	0				0.9	0.04576	0.9	0.04576	2	1	1
46	1	0	0	1	3100	1	1		2.9	0					2.9		2.9	2	1	0
76	1	1	0	1	5	0	1	0.2	0.69897	0				0.9	0.04576	1.2	-0.0792	2	1	1
64	1	1	0	1	1	0	0		2.9	0					2.9		2.9	2	1	0
85	1	1	1	0		0	0		2.6	0				0.1	1	0.3	0.52288	2	1	1
85	1	0	1	0		0	0	0.01	2	0				0.03	1.52288	0.02	1.69897	2	1	0
86	1	1	0	2	600	1	1		2.6	0				0.1	1	0.3	0.52288	0	1	1
78	1	1	1	1		1	1		2.9	0					2.9		2.9	2	1	0
81	1	1	0	1	1980	0	1	0.01	2	0				0.8	0.09691	0.9	0.04576	2	1	1
67	0	1	0	1	33	0	0	0.9	0.04576	0				1	0	1.2	-0.0792	2	1	1
70	0	0	0	1	1	1	1		2.6	0				0.1	1	0.3	0.52288	2	1	1
44	1	1	0	1	14600	1	1		2.3	0				0.03	1.52288	0.03	1.52288	2	1	0
54	1	1	0	9	1400	1	1		2.9						2.9		2.9	2	0	0
76	0	0	0	1	3	0	1		2.3	0				0.1	1	0.6	0.22185	2	1	1
70	1	0	0	1	1170	0	1	0.4	0.39794					0.7	0.1549	0.9	0.04576	0	1	1
70	0	0	0	0	0	0	0	0.04	1.39794	0				0.2	0.69897	0.2	0.69897	2	1	1
88	1	0	0	0	0	1	1		2.6	1	Streptoco	3		0.01	2	0.6	0.22185	2	1	1
65	0	0	0	0	6	1	1	0.2	0.69897	1	Methicill	1		0.8	0.09691	0.7	0.1549	2	1	1
78	1	1	0	3	8	1	1		2.3	1	Methicill	1		0.08	1.09691	0.15	0.82391	2	1	1
84	1	1	0	1	6	1	1		2.6	1	Methicill	2			2.6		2	2	1	0
80	1	1	0	5	69	1	1	0.01	2	1	Staphyloc	1		0.7	0.1549	0.9	0.04576	2	1	1
63	1	0	0	19	864	1	1		2	1	Streptoco	3		0.2	0.69897	0.2	0.69897	2	1	1
58	0	1	1	14		1	1	0.6	0.22185	1	Candida :	8	1	1.5	-0.1761	1.2	-0.0792	2	1	1
69	0	0	0	0	2	1	1		2	1	Haemoph	7		0.7	0.1549	1	0	2	1	1
44	0	0	0	1	11	1	1		2	1	Methicill	1		0.06	1.22185	0.03	1.52288	2	1	0
75	0	1	0	1	44	1	1		2.9	1	Streptoco	3			2.9		2.9	2	1	0
58	0	1	0	2	2	1	1		2.6	1	Rahnella	5		0.04	1.39794	0.3	0.52288	2	1	1
77	1	0	0	1	17	1	1		2	1	Methicill	1		0.7	0.1549	1	0	2	1	1
69	0	0	0	1	4	1	1		2.3	1	Methicill	1		0.13	0.90309	0.08	1.09691	2	1	0
61	0	0	0	5	1523	1	1	0.7	0.1549	0				0.6	0.22185	0.7	0.1549	2	1	1
83	0	1	0	5	18	0	1	0.01	2	1	Aspergill	8	0	0.04	1.39794	0.07	1.1549	2	1	0
72	0	1	0	2	3	0	1		2.3	1	Staphyloc	1	0	0.1	1	0.1	1	2	1	1
63	0	1	0	1	1	1	1		2	1	Moraxell	7	0		2.9		2.9	2	1	0
85	1	1	0	0	3	0	1		2.3	1	Methicill	2	0	0.1	1	0.1	1	2	1	1
85	1	1	0	10	26	0	0		2.3	1	Methicill	1	0	0.1	1	0.1	1	2	1	1
86	1	0	1	5		0	0		2.3	1	Klebsiell:	5	1		2.3	0.8	0.09691	2	1	1
86	1	1	1	5		0	0		2.6	1	Klebsiell:	5	1		2.3		2.9	2	1	0
77	0	0	1	7		0	0		2	1	Magnusia	8	0	0.9	0.04576	0.1	1	2	1	1
77	1	1	0	2	7	0	1		2.3	1	Methicill	1	0	0.2	0.69897	0.6	0.22185	2	1	1
80	1	0	0	4	2920	0	1	0.05	1.30103	0				0.15	0.82391	0.15	0.82391	2	1	1
79	0	1	0	0	6	0	1	1	0	0				1	0	1.5	-0.1761	2	1	1
71	0	0	0	7	990	0	1	0.06	1.22185	1	Neisseria	7	0	0.3	0.52288	0.3	0.52288	2	1	1
74	0	0	0	0	450	1	0	0.9	0.04576	0				0.4	0.39794	0.6	0.22185	2	1	1
75	0	0	0	0	4	0	0	0.4	0.39794	0				0.9	0.04576	1	0	2	1	1
80	0	1	1	30		0	1	0.3	0.52288	1	Candida :	8	1	0.06	1.22185	0.1	1	2	1	1
71	1	0	0	5	5	1	0	0.2	0.69897	1	Staphyloc	2		0.8	0.09691	0.9	0.04576	2	1	1
76	0	0	0	2	2	1	1	0.08	1.1	0				0.15	0.82391	0.2	0.69897	2	1	1
62	1	1	0	1	1	1	0	0.1	1	0				0.7	0.1549	0.7	0.1549	2	1	1
86	1	1	0	4	7	0	0		2.6	1	Methicill	2			2.9		2.9	2	0	0
72	1	1	0	1	3	1	1	0.3	0.52288	0				0.01	2		2.3	2	1	0
43	1	1	1			0	0	0.06	1.22185				Candida :	0.05	1.30103	0.05	1.30103	0	1	0
78	1	1	0	3	1460	0	0		2.3	1	Streptoco	3		0.05	1.30103	0.2	0.69897	2	1	1
83	1	1	0	0	1825	1	1		2.6	1	Leuconos	7		0.15	0.82391	0.3	0.52288	2	1	1
89	0	1	0	2	3	1	1	0.08	1.1	0				0.1	1	0.3	0.52288	2	1	1
30	0	1	0	2	2	1	1		2.6		Staphyloc	1		0.5	0.30103	1.2	-0.0792	2	1	1
68	0	0	0	2		1	1		2.6	0					2.9		2.9	2	0	0
73	1	1	0	10		1	1		2.6	1	Candida :	8			2.6		2.6	2	0	0
87	0	1	0	1	7	1	0	0.3	0.52288	0				0.3	0.52288	0.4	0.39794	2	0	1
78	1	1	0	10	10	1	1	0.05	1.30103	1	Fusarium	8			2.9		2.9	2	0	0
74	0	1	1	0			1	1.2	-0.0792				Candida :	1.2	-0.0792	1.5	-0.1761	0	0	1
83	0	0	0	4	365	1	1	0.04	1.39794	0					2.3	0.02	1.69897	2	0	0
82	1	0	0	6	6	0	1	0.02	1.69897	1	Staphyloc	2		0.4	0.39794	0.5	0.30103	2	0	1
46	0	0	0	1	1	1	1		2.3					0.08	1.09691	0.1	1	2	0	1
70	1	0	0	15	15	0	1	0.2	0.69897	0				0.4	0.39794	0.9	0.04576	2	0	1
56	1	0	0	35	35	0	1	0.8	0.09691					1	0	1.2	-0.0792	0	0	1
61	0	1	0	0	11	0	0	1	0	0				1.2	-0.0792	1.2	-0.0792	0	1	1
89	0	0	1	1		0	0	0.3	0.52288	0			0	0.7	0.1549	0.9	0.04576	0	1	1
89	0	1	1	1		0	0	0.1	1	0			0	0.2	0.69897	0.3	0.52288	0	1	1
82	1	0	0	14	28	0	0		2	0				0.3	0.52288	0.8	0.09691	2	1	1
19	1	1	0	1	5	1	0	0.5	0.30103	0				0.6	0.22185	0.7	0.1549	2	1	1
70	1	0	0	1	3	0	0		2.3	1	Methicill	1		0.05	1.30103	0.05	1.30103	2	1	0
66	0	0	0	0	70	0	0	0.4	0.39794	0				0.9	0.04576	0.9	0.04576	2	1	1
65	0	1	0	0	2	0	0		2.6	1	Methicill	1		0.4	0.39794	0.9	0.04576	2	1	1
73	0	0	0	1	3650	1	0		2	0				0.5	0.30103	0.7	0.1549	2	1	1
33	0	1	0	7	7	0	1		2.3	0				0.2	0.69897	0.6	0.22185	2	1	1
49	0	0	0	1	4380	0	1	0.2	0.69897	0				1.2	-0.0792	1.2	-0.0792	2	1	1
81	0	0	0	0	6	0	0	1	0	0				0.7	0.1549	1.2	-0.0792	0	1	1
50	0	0	1	0		0	0	0.8	0.09691	0				1.2	-0.0792	1.2	-0.0792	0	1	1
57	1	0	0	0	2	1	1		2.3	0				0.9	0.04576	1.2	-0.0792	2	1	1
36	0	1	0	0	6	0	1	0.02	1.69897	0				0.3	0.52288	0.3	0.52288	2	1	1
65	0	0	0	0	2	0	1		2.9	1	Enterococ	6			2.9		2.9	2	1	0
62	1	0	0	0	46	0	0	0.01	2	0				0.01	2	0.02	1.69897	2	1	0
84	1	0	0	0	2	0	0	0.02	1.69897	0				0.3	0.52288	0.4	0.39794	2	1	1
83	1	1	0	0	1	0	0	0.3	0.52288	0				0.7	0.1549	0.9	0.04576	1	1	1
65	1	1	0	0	4	0	0		2	0				0.1	1	0.15	0.82391	2	1	1
66	0	1	0	1	4	0	0		2.3	1	Staphyloc	1		0.4	0.39794	0.3	0.52288	2	1	1
81	0	0	0	0	3	0	0		2.6	0				0.15	0.82391	0.1	1	2	1	1
82	1	1	0	14	82	0	0		2.3	0				0.02	1.69897	0.08	1.09691	1	1	0
91	0	1	0	2	12	1	0		2.3	1	Methicill	1		0.5	0.30103	0.3	0.52288	2	1	1
87	1	1	0	4	5	1	0		2.6	1	Enterococ	6			2.3		2.6	2	1	0
79	0	1	0	0	3	0	0		2.3	1	Methicill	1		0.1	1	0.15	0.82391	2	1	1
57	0	0	0	0	4	0	0	1.2	-0.0792	0				1.2	-0.0792	1.2	-0.0792	2	1	1
75	1	1	0	0	1	0	1	0.3	0.52288	1	Coryneba	7		1.2	-0.0792	1.2	-0.0792	2	1	1
75	1	1	0	8	8	1	1		2.3	1	Methicill	2			2.3	0.01	2	0	1	0
61	0	1	0	1	1	1	1		2.3	0				0.07	1.1549	0.07	1.1549	2	1	0
25	0	1	0	1	7	1	1		2.3	1	Methicill	2		0.3	0.52288	0.4	0.39794	2	1	1
74	1	0	0	0	5	0	0		2.3	1	Methicill	1		0.3	0.52288	1	0	2	1	1
23	0	1	0	0	0	1	1	0.05	1.30103	0				0.8	0.09691	0.8	0.09691	2	1	1
46	1	0	1	1		0	0	0.01	2	0				0.15	0.82391	0.15	0.82391	2	1	1
57	0	1	0	0	11	0	0	0.04	1.39794	0				0.4	0.39794	0.3	0.52288	2	1	1
77	1	1	0	7	45	1	0	0.4	0.39794	0				0.9	0.04576	0.9	0.04576	0	1	1
59	1	1	0	1	5	0	0		2.3	1	Staphyloc	1		0.15	0.82391	0.4	0.39794	2	1	1
54	0	0	1	7		0	0	0.04	1.39794	1	Candida :	8	0	0.4	0.39794	0.7	0.1549	2	1	1
54	0	1	1	7		0	0	0.02	1.69897	1	Candida :	8	0	0.07	1.1549	0.1	1	2	1	1
81	1	0	0	0	0	1	0	0.1	1	1	Methicill	1		0.07	1.1549	0.08	1.09691	2	1	0
78	1	0	1	1		0	0	0.2	0.69897	0				0.5	0.30103	0.8	0.09691	2	1	1
78	1	1	1	1		0	0		2.3	0				0.1	1	0.2	0.69897	2	1	1
73	0	0	1	1		0	0		2	0				0.2	0.69897	0.3	0.52288	2	1	1
73	0	1	1	1		0	0		2.3	0				0.4	0.39794	0.7	0.1549	2	1	1
57	0	1	1	2		1	1	1	0	0				1.2	-0.0792	1.2	-0.0792	0	1	1
68	1	0	1	11		0	0		2	1	Staphyloc	2	1	0.4	0.39794	0.8	0.09691	2	1	1
68	1	1	1	11		0	0	0.01	2	1	Staphyloc	2	1	0.4	0.39794	0.9	0.04576	2	1	1
77	1	1	0	1	18	0	0	0.07	1.1549	0				0.5	0.30103	0.7	0.1549	0	1	1
79	1	0	0	2	6	0	0	0.02	1.7	0				0.4	0.39794	0.6	0.22185	2	1	1
53	0	1	0	3	11	1	1		2	0				0.08	1.09691	0.08	1.09691	2	1	0
66	0	1	0	3	9125	0	1		2.3	0				0.15	0.82391	0.4	0.39794	2	1	1
54	0	0	1	12		0	0	0.02	1.69897	0				0.2	0.69897	0.15	0.82391	2	1	1
54	0	1	1	12		0	0	0.04	1.39794	0				0.01	2	0.4	0.39794	2	1	1
56	0	0	0	21		1	1	0.7	0.1549	0				0.7	0.1549	1.2	-0.0792	1	1	1
55	0	1	0	5	14235	1	1		2.3	0					2.6		2.6	1	1	0
77	0	1	0	0		1	1		2.6	1	Streptoco	4			2	0.03	1.52288	2	1	0
56	0	0	0	1	1825	0	1	0.5	0.30103	0				0.5	0.30103	0.6	0.22185	0	1	1
69	1	0	1	30		0	0	0.7	0.1549	1	Candida :	8	1	1	0	1	0	2	1	1
69	1	1	1	30		0	0	0.7	0.1549	1	Candida :	8	1	0.7	0.1549	1	0	2	1	1
78	0	0	0	0	30	0	0	0.4	0.39794	0				0.4	0.39794	0.7	0.1549	2	1	1
78	0	1	0	0	30	0	0	0.7	0.1549	0				0.8	0.09691	1	0	2	1	1
79	0	1	0	7	100	0	0	0.4	0.4	1	Purpureo	8			2	0.02	1.69897	2	1	0
76	1	1	0	1	4	0	0		2.3	0				0.2	0.69897	0.2	0.69897	2	1	1
77	1	1	0	1	19	0	0	0.6	0.22185	1	Purpureo	8		0.9	0.04576	0.4	0.39794	2	1	1
75	0	0	0	7	24	1	1	0.01	2	1	Staphyloc	1		0.4	0.39794	0.5	0.30103	2	1	1
68	1	1	0	0.5	1	1	1	0.4	0.39794	0				0.09	1.04576	0.1	1	2	1	1
79	1	1	0	0.5	1	1	1		2.3	0				0.2	0.69897	0.4	0.39794	2	1	1
61	1	1	0	1	1	1	1		2.6	0					2.6		2.6	2	1	0
40	1	1	0		60		1	0.05	1.30103	0					2.3	0.1	1	2	1	1
69	1	1	0	0.5	3	1	1	0.6	0.22185	1	Staphyloc	1	0	0.8	0.09691	0.7	0.1549	2	1	1
77	0	0	0	4	4	1	1		2.3	1	Staphyloc	1	0	0.5	0.30103	0.9	0.04576	2	1	1
53	0	1	0	1	3	1	1	0.06	1.2	0				0.15	0.82391	0.2	0.69897	2	1	1
82	1	1	0	2	700	1	1	0.1	1	0				0.3	0.52288	0.7	0.1549	2	1	1
66	1	1	0	0.5	1	1	1	0.05	1.30103	0				0.2	0.69897	0.4	0.39794	2	1	1
75	1	1	0	0.5	3	1	1		2.3	0				0.4	0.39794	0.4	0.39794	2	1	1
72	0	0	1	1		1	1	0.15	0.82391	0				1.2	-0.0792	1.2	-0.0792	2	1	1
72	1	0	0	4		1	1		2	0				0.04	1.39794	0.15	0.82391		1	1
69	0	1	1	4		0	1		2.3	1	Streptoco	3	0		2.3		2.3	2	1	0
80	1	1	0	0.5	1.5	1	1		2.3	1	Enterococ	6	0	0.05	1.30103	0.01	2	2	1	0
66	1	0	1	3		1	1		2.3	1	Streptoco	3	0	0.1	1	0.2	0.69897	2	1	1
66	1	1	1	3		1	1		2.9	1	Streptoco	3	0		2.9		2.9	2	1	0
87	1	1	0	30	150	0	1	0.01	2	0				0.05	1.30103	0.2	0.69897	1	1	1
79	0	1	0	0.5	3	1	1	0.01	2	1	Bacillus :	7	0	0.1	1	0.08	1.09691	2	1	0
66	1	1	0	0.5	3	1	1	0.02	1.69897	0				0.15	0.82391	0.2	0.69897	2	1	1
85	1	1	0	7	700	1	1		2.6	0				0.03	1.52288	0.03	1.52288	2	1	0
86	1	0	1	50		1	1	0.04	1.39794	0				0.4	0.39794	0.5	0.30103	2	1	1
86	1	1	1	50		1	1	0.06	1.22185	0				0.04	1.39794		2.9	2	0	0
6	0	1	0	1	10	1	1		2.3	1	Streptoco	4	0		2.6		2.6	2	1	0
73	1	0	0	0.5	2	0	1	0.4	0.39794	0				0.4	0.39794	0.5	0.30103	2	1	1
76	1	0	0	1	20	0	1		2.3	0				0.3	0.52288	0.2	0.69897	2	1	1
83	0	1	1	4		1	1		2.3	0				0.1	1	0.1	1	2	1	1
79	1	0	0	0.5	3	1	1	0.15	0.82391	0				0.1	1	0.3	0.52288	2	1	1
78	1	1	0	1	3	1	1	0.3	0.52288	0				0.6	0.22185	0.6	0.22185	2	1	1
74	1	1	0	1	5	1	1		2.3	1	Staphyloc	1	0	0.2	0.69897	0.4	0.39794	2	1	1
78	0	1	0		14	0	1		2.6	0				0.02	1.69897	0.02	1.69897	2	1	0
38	0	0	1	5		1	1		2.6	1	Enterobac	5	0		2.3		2.6	2	1	0
46	0	1	0	1	2	1	1		2.3	0					2.3		2.3	2	1	0
79	1	0	0	3	7	1	1	0.1	1	0				0.1	1	0.09	1.04576	2	1	0
73	0	0	0	2	120	1	1		2	0				0.02	1.69897	0.15	0.82391	2	1	1
70	1	1	0	0.5	1	0	0		2.3	0				0.3	0.52288	0.3	0.52288	2	1	1
73	1	1	1	4		1	1		2.3	0					2	0.02	1.69897	2	1	0
17	1	1	0	1	3	0	1	0.2	0.69897	1	Staphyloc	1	0	0.9	0.04576	1	0	2	1	1
58	1	1	0	1	6	1	1	0.01	2	0				0.3	0.52288	0.8	0.09691	2	1	1
57	1	0	1	5		1	1	0.01	2	0				0.8	0.09691	0.4	0.39794	2	1	1
67	1	0	0	0.5	3	1	1	0.1	1	0				0.5	0.30103	1.2	-0.0792	2	1	1
90	1	1	1	3		1	1		2.6	1	Staphyloc	2	0	0.05	1.30103	0.08	1.09691	2	1	0
72	1	0	0	1	1	0	0	0.2	0.69897	0				0.2	0.69897	0.4	0.39794	0	1	1
61	0	0	0	1	4	0	1		2.3	0				0.4	0.39794	1	0	0	1	1
62	0	0	1	1		0	0	0.1	0.1549	0				0.3	0.52288	0.6	0.22185	1	1	1
62	0	1	1			0	0	1	0	0				0.2	0.69897	1.5	-0.1761	1	1	1
46	0	1	1			0	0	1	0					1	0	0.9	0.04576	0	1	1
46	0	0	1			0	0	1	0					1	0	1	0	0	1	1
69	1	1	1	2		0	0	1	0					1	0	1	0	1	1	1
69	1	0	1	2		0	0	1	0					1	0	1.2	-0.0792	1	1	1
71	1	1	0	2	2	1	0		2.6	0					2	0.01	2	2	1	0
76	0	0	0	10	440	0	0		2.3	1	Coryneba	7	0	0.01	2	0.01	2	2	1	0
74	0	0	0	2	2591	1	0		2.3	0				0.04	1.39794	0.05	1.30103	2	1	0
80	1	1	0	1	1	1	0	0.08	1.1	0				0.15	0.82391	0.1	1	2	1	1
81	0	1	0	8	35	0	0		2.3	0					2.3	0.1	1	2	1	1
62	0	1	0	3	2016	1	1	0.4	0.39794	0				0.4	0.39794	0.2	0.69897	1	1	1
88	1	1	0	2	1460	1	1	0.01	2	0				0.3	0.52288	0.4	0.39794	2	1	1
89	1	1	0	1	3073	1	0	0.4	0.39794					0.5	0.30103	0.5	0.30103	1	1	1
48	0	1	0	31	730	1	0	0.8	0.09691	0				0.1	1	0.6	0.22185	1	1	1
52	1	0	0	1	10	1	0	0.04	1.39794	1	Methicill	1		1	0	0.7	0.1549	1	1	1
72	1	1	0	5	92	0	0	0.6	0.22185	0				0.9	0.04576	0.8	0.09691	1	1	1
59	1	0	1	9		0	0	0.4	0.39794					1	0	0.9	0.04576	0	1	1
55	0	0	0	2	2	1	0		2.3	0				0.3	0.52288	0.9	0.04576	2	1	1
69	1	0	0	1	7	1	0	0.4	0.39794	0				0.4	0.39794	1	0	2	1	1
82	1	1	0	3	1460	0	0	0.3	0.52288	0				0.5	0.30103	1	0	0	1	1
47	1	0	0	1	4	0	0	0.8	0.1	0				0.9	0.04576	1.2	-0.0792	2	1	1
71	0	1	0	1	1	0	0		2	0				1	0	1.2	-0.0792	2	1	1
51	1	0	0	1	8	0	1		2	0				1.5	-0.1761	1.5	-0.1761	2	1	1
67	0	0	0	3		1	1		2.3	0				1	0	1.5	-0.1761	2	1	1
69	0	0	0	2	2	1	1		2.3	1	Streptoco	4	0		2.9		2.9	2	1	0
80	0	1	0	4	7	1	0		2.6	0					2.9		2.9	1	1	0
83	1	1	0	2	2158	1	0		2.6	1	Streptoco	4	0		2.9		2.9	1	0	0
77	0	0	0	5	103	1	0		2.9	0					2.9		2.9	1	1	0
65	0	1	1	1		0	0		2.9	1	Bacillus :	7	1		2.9		2.9	1	0	0
85	1	1	0	1	4380	1	1		2.9	1	Streptoco	3			2.9		2.9	2	0	0
71	1	1	0	1	3	0	0		2.6	1	Enterococ	6			2.6		2.3	2	1	0
78	0	1	0	2	7	1	1		2.3	0				0.03	1.52288	0.03	1.52288	2	1	0
43	0	0	1	1		1	1	0.01	2	1	Staphyloc	2	1	0.01	2	0.01	2	2	1	0
73	1	1	0	2	30	0	1	0.01	2	0				0.5	0.30103	0.5	0.30103	2	1	1
84	1	0	0	1	7	0	1	0.04	1.39794	0				0.1	1	0.1	1	2	1	1
53	1	0	0	1	2	1	1		2.3	0				0.5	0.30103	0.6	0.22185	2	1	1
70	1	1	0	2	90	0	1	0.03	1.52288	0				0.4	0.39794	0.8	0.09691	2	1	1
72	0	1	0	1	1	1	1	0.5	0.30103	0				0.6	0.22185	0.8	0.09691	2	1	1
80	1	0	1	18		1	1		2.6	0				0.4	0.39794	0.8	0.09691	2	1	1
44	0	1	0	2	3	1	1		2.6	1	Staphyloc	1	0	0.1	1	0.4	0.39794	2	1	1
57	1	0	0	40	40	1	1	0.01	2	0					2.3		2.3	2	1	0
76	0	0	1	1		1	1	0.01	2	1	Methicill	2	1	1	0	1	0	2	1	1
78	1	1	0	1	1	1	1		2.6	0				0.4	0.39794	0.8	0.09691	2	1	1
71	0	0	0	4	4	1	1	0.01	2	0				0.2	0.69897	0.2	0.69897	2	1	1
76	1	1	0	2	3	0	1	0.03	1.52288	0				0.8	0.09691	0.8	0.09691	2	1	1
83	0	1	0	3	39	0	1		2.3	0				0.1	1	0.1	1	2	1	1
71	1	0	0	1	9	0	1		2.3	0				0.3	0.52288	0.4	0.39794	2	1	1
64	0	0	0	1	1	1	1		2.3	1	Staphyloc	1	0	0.1	1	0.4	0.39794	2	1	1
54	1	1	1	1		0	1	0.02	1.69897	0				0.01	2	0.01	2	2	1	1
68	1	0	0	17	90	0	1	0.8	0.09691	1	Penicilliu	8	0	1	0	1	0	2	1	1
43	1	1	0	1	1	1	1	0.01	2	1	Bacillus :	7	0		2.9		2.9	2	1	0
83	1	0	0	1	2	0	1		2.3	1	Coryneba	7	0	0.06	1.22185	0.4	0.39794	2	1	1
73	1	0	1	10		0	1	1	0	1	Candida :	8	1	1	0	1	0	2	1	1
73	1	1	1	10		0	1	1	0	1	Candida :	8	1	1	0	1	0	2	1	1
78	0	1	0	298	298	1	1	0.01	2	1	Paecilom	8	0		2.9		2.9	2	0	0
80	0	1	1	7		1	1		2.6	0					2.9		2.9	2	1	0
70	1	1	1	3		1	1		2.3	1	Aspergill	8	0	0.1	1	0.2	0.69897	2	1	1
57	1	0	0	1	1	0	1	0.5	0.30103	0				0.4	0.39794	0.4	0.39794	2	1	1
75	0	1	1	1		0	1	0.2	0.69897	1	Methicill	2	1	0.5	0.30103	0.4	0.39794	2	1	1
69	0	0	1	6		0	1	0.2	0.69897	0				0.8	0.09691	1.2	-0.0792	2	1	1
69	0	1	1	6		0	1	0.2	0.69897	0				0.4	0.39794	0.8	0.09691	2	1	1
78	1	1	1	1		1	1		2.6	1	Klebsiell:	5	1		2.9		2.9	2	0	0
69	1	0	0	2	2	1	1		2.6	0					2.3		2.9	2	1	0
79	0	1	0	1	5	0	0		2.3	0				0.09	1.04576	0.1	1	2	1	1
74	1	1	0	0	2	1	1	0.6	0.22	0				0.7	0.1549	0.8	0.09691	2	1	1
45	1	1	1	20		0	0	0.2	0.69897				Candida :	1	0	1	0	0	1	1
91	1	1	1	6		0	0		2.6	1	Klebsiell:	5	0		2.6		2.6	2	1	0
67	0	0	0	0	8	0	0		2	1	Streptoco	4	0	1	0	1.2	-0.0792	2	1	1
72	1	0	0	1	4	0	0		2	0				0.4	0.39794	0.4	0.39794	2	1	1
67	0	0	0	2	27	0	0		2	0				1	0	1.2	-0.0792	2	1	1
37	0	0	0	1	2	0	0	0.01	2	1	Methicill	1	0	1.5	-0.1761	1.5	-0.1761	2	1	1
77	1	1	0	1	7	0	0		2	0				0.9	0.04576	1.2	-0.0792	2	1	1
69	1	1	1	4		1	1		2.3	0				0.8	0.09691	0.4	0.39794	2	1	1
72	1	1	1	2		1	0		2.3	0				0.15	0.82391	0.3	0.52288	2	1	1
70	1	1	0	0	4	0	0		2.3	0				0.7	0.1549	0.8	0.09691	2	1	1
75	1	0	0	0	120	1	0	0.9	0.04576	0				0.15	0.82391	0.2	0.69897	2	1	1
78	1	0	1	2		1	1		2.9	1	Streptoco	4	0		2.9		2.9	2	1	0
81	1	1	0	0	3	0	1		2.3	0					2	0.2	0.69897	2	1	1
47	0	1	0	3	9	1	0	0.7	0.1549	1	Methicill	1	0	0.7	0.1549	1.2	-0.0792	2	1	1
59	0	1	0	6	20	1	0		2.3	0				0.3	0.52288	0.1	1	2	1	1
58	1	0	0	1	2	1	0		2.3	1	Methicill	1	0	0.1	1	0.2	0.69897	2	1	1
75	0	1	0	1	2310	1	1		2.6	1	Streptoco	3	0		2.6		2.9	2	1	0
65	0	1	0		36			0.08	1.09691	1	Staphyloc	1		0.3	0.52288	0.3	0.52288	2	1	1
46	0	0	1	6		0	0		2.3	1	Methicill	2	1	0.2	0.69897	0.2	0.69897	2	1	1
59	0	0	1	1		0	0	0.03	1.52288	1	Streptoco	3	1		2.9		2.9	2	1	0
81	0	0	0	4	20	1	0	0.2	0.69897	0				0.4	0.39794	1	0	2	1	1
94	0	1	0	0	7	0	0		2	0				0.7	0.1549	0.7	0.1549	2	1	1
85	0	1	0	1	3	0	0	0.05	1.3	1	Staphyloc	1	0	0.3	0.52288	0.4	0.39794	2	1	1
69	0	0	1			0	0	0.02	1.69897	1	Candida :	8	1		2.6		2.9	2	1	0
91	1	1	0	3	45	0	0		2	0				0.1	1	0.1	1	2	1	1
70	1	1	0	0	4	0	0		2.3	0				0.8	0.09691	0.8	0.09691	2	1	1
56	0	1	0	1	34	0	0		2	1	Staphyloc	2	0	0.1	1	0.2	0.69897	2	1	1
84	1	1	0	5	5	0	0	0.3	0.52288	0				0.06	1.22185	0.3	0.52288	2	1	1
84	1	1	0	8	43	0	1	0.5	0.30103	0				0.8	0.09691	0.6	0.22185	2	1	1
92	1	0	0	2	2	0	0	0.3	0.52288	0				0.4	0.39794	0.6	0.22185	0	1	1
76	0	0	0	1	11	1	0	0.2	0.69897	0				0.7	0.1549	0.8	0.09691	1	1	1
69	1	1	0	1	4	0	0	0.1	1	1	Staphyloc	1		0.2	0.69897	0.08	1.09691	2	1	0
90	0	1	0	20	50	0	1	0.4	0.39794	0				0.7	0.1549	0.7	0.1549	1	1	1
87	1	1	0	1	5	0	1	0.5	0.3	0				0.8	0.09691	0.9	0.04576	0	1	1
78	1	1	0	1	1	0	1	1	0	0				1	0	1	0	0	1	1
84	0	0	0	3	6	0	1		2.3	0				0.15	0.82391	0.15	0.82391	2	1	1
76	0	0	0	2	2555	1	1		2.6	0					2.9		2.9	2	1	0
81	0	1	0	1	6	0	0		2.3	0				0.08	1.09691	0.2	0.69897	2	1	0
76	0	0	0	10	32	0	0	0.05	1.30103	1	Staphyloc	1	0	0.07	1.1549	0.15	0.82391	2	1	1
94	0	0	0	3	4745	1	1		2.9	0					2.9		2.9	0	1	0
78	0	1	0	1	2	0	0	0.5	0.30103	0				0.1	1	0.2	0.69897	2	1	1
72	0	1	0	2	5	0	0	0.2	0.69897	0				0.7	0.1549	0.8	0.09691	0	1	1
57	1	0	1	3		0	1	0.03	1.52288	0				0.1	1	0.15	0.82391	2	1	1
57	1	1	1	3		0	1	0.08	1.09691	0				0.3	0.52	0.4	0.39794	2	1	1
76	1	0	1	1		0	1	0.6	0.22185	0				1	0	1.2	-0.0792	2	1	1
79	0	0	1	3		0	0	1	0	0				0.8	0.09691	0.9	0.04576	0	1	1
79	0	1	1	3		0	0	0.4	0.39794	0				0.5	0.30103	0.5	0.30103	0	1	1
77	1	0	0	6	31	0	1	1.2	-0.08	0				1.2	-0.08	1.2	-0.08	2	1	1
60	1	1	0	7	1	0	0	0.01	2	0				1.2	-0.08	1.2	-0.08	2	1	1
84	1	0	0	0	2	1	1	0.5	0.3	0				1.2	-0.08	1.2	-0.08	2	1	1
79	1	0	0	4	11	0	1	0.01	2	0				0.3	0.52	0.3	0.52	2	1	1
70	0	1	0	6	66	1	1	HM	2.3	0				0.6	0.22	0.5	0.3	2	1	1
87	1	1	0	1	3	0	0	HM	2.3	1	Staphyloc	1		0.2	0.7	0.2	0.7	2	1	1
68	0	0	0	1	91	0	0	0.07	1.15	0				0.5	0.3	0.8	0.1	2	1	1
75	0	0	0	1	3	0	0	HM	2.3	0				0.04	1.4	0.1	1	2	1	1
65	1	1	0	3	7	1	1	0.3	0.52	0				0.5	0.3	0.8	0.1	2	1	1
72	0	1	0	0	3	1	1	0.5	0.3	1	Staphyloc	1		0.5	0.3	0.8	0.1	2	1	1
50	0	0	1	4		0	0	HM	2.3	0			0	0.04	1.4	0.01	2	2	1	0
79	1	1	0	3	9	0	0	HM	2.3	0				0.7	0.15	0.9	0.05	2	1	1
