# Supplementary material for: Clinical Characteristics and Outcomes in 314 Japanese Patients with Bacterial Endophthalmitis: A Multicenter Cohort Study from J-CREST
Source: Pathogens. 2021 Mar 24;10(4):390. doi: 10.3390/pathogens10040390 (PMC8063932; doi:10.3390/pathogens10040390)
Supplement: Supplementary file 1 [file Table S1-1.pdf › pathogens-1151745 supplementary- proof/Table S1-1.pdf]

Age	Sex: M=0	Mono eye	Type of en
77	0	0	1
32	1	0	2
56	1	1	2
57	0	0	2
36	1	0	2
69	0	1	2
76	0	0	2
43	0	1	2
44	1	1	2
62	0	0	1
70	0	0	1
48	1	0	2
76	0	0	1
47	0	0	1
82	0	1	2
9	0	0	1
60	0	1	2
74	1	1	2
51	0	1	2
40	1	1	2
58	0	0	1
73	0	0	1
81	1	0	1
58	0	0	2
55	1	0	2
64	0	0	2
55	1	1	2
45	0	0	2
78	1	0	1
10	1	0	1
67	1	0	2
63	0	0	1
45	1	1	2
88	1	1	2
63	0	0	2
81	0	0	1
84	1	0	1
75	1	0	1
63	0	0	1
60	0	1	2
84	0	0	1
46	1	0	1
76	1	0	1
64	1	0	1
85	1	1	2
86	1	0	1
78	1	0	2
81	1	0	1
67	0	0	1
70	0	0	1
44	1	0	1
54	1	0	1
76	0	0	1
70	0	1	1
88	1	0	1
65	0	0	1
78	1	0	1
84	1	0	1
80	1	0	1
63	1	0	1
58	0	0	2
69	0	0	1
44	0	0	1
75	0	0	1
58	0	0	1
77	1	0	1
69	0	0	1
61	0	0	1
83	0	0	1
72	0	0	1
63	0	0	1
85	1	1	1
86	1	1	2
77	0	1	2
80	1	0	1
79	0	0	1
71	0	0	1
74	0	0	1
75	0	0	1
80	0	0	2
71	1	0	1
76	0	0	1
62	1	0	1
86	1	0	1
72	1	0	1
43	1	0	2
78	1	0	1
83	1	0	1
89	0	0	1
30	0	0	1
68	0	0	1
73	1	0	1
87	0	0	1
78	1	0	1
74	0	0	2
83	0	0	1
82	1	0	1
46	0	0	1
70	1	0	1
56	1	0	1
61	0	0	1
89	0	1	2
82	1	0	1
19	1	0	1
70	1	0	1
66	0	0	1
65	0	0	1
73	0	0	1
33	0	0	1
49	0	0	1
81	0	0	1
50	0	0	2
57	1	0	1
36	0	0	1
65	0	0	1
62	1	0	1
84	1	0	1
83	1	0	1
65	1	0	1
66	0	0	1
81	0	0	1
82	1	0	1
91	0	0	1
87	1	0	1
79	0	0	1
57	0	0	1
75	1	0	1
75	1	0	1
61	0	0	1
25	0	0	1
74	1	0	1
23	0	0	1
46	1	0	2
57	0	0	1
77	1	0	1
59	1	0	1
54	0	1	2
81	1	0	1
78	1	1	2
73	0	1	2
57	0	0	2
68	1	1	2
77	1	0	1
79	1	0	1
53	0	0	1
66	0	0	1
54	0	1	2
56	0	0	1
55	0	0	1
77	0	0	1
56	0	0	1
69	1	1	2
78	0	1	1
79	0	0	1
76	1	0	1
77	1	0	1
75	0	0	1
68	1	0	1
79	1	0	1
61	1	0	1
40	1	0	1
69	1	0	1
77	0	0	1
53	0	0	1
82	1	0	1
66	1	0	1
75	1	0	1
72	0	1	2
69	0	0	2
80	1	0	1
66	1	1	2
87	1	0	1
79	0	0	1
66	1	0	1
85	1	0	1
86	1	1	2
6	0	0	1
73	1	0	1
76	1	0	1
83	0	0	2
79	1	0	1
78	1	0	1
74	1	0	1
78	0	0	1
38	0	0	2
46	0	0	1
79	1	0	1
73	0	0	1
70	1	0	1
73	1	0	2
17	1	0	1
58	1	0	1
57	1	0	2
67	1	0	1
90	1	0	2
72	1	0	1
61	0	0	1
62	0	1	2
46	0	1	2
69	1	1	2
71	1	0	1
76	0	0	1
74	0	0	1
80	1	0	1
81	0	0	1
62	0	0	1
88	1	0	1
89	1	0	1
48	0	0	1
52	1	0	1
72	1	0	1
59	1	0	2
55	0	0	1
69	1	0	1
82	1	0	1
47	1	0	1
71	0	0	1
51	1	0	1
67	0	0	1
69	0	0	1
80	0	0	1
83	1	0	1
77	0	0	1
65	0	0	2
85	1	0	1
71	1	0	1
78	0	0	1
43	0	0	2
73	1	0	1
84	1	0	1
53	1	0	1
70	1	0	1
72	0	0	1
80	1	0	2
44	0	0	1
57	1	0	1
76	0	0	2
78	1	0	1
71	0	0	1
76	1	0	1
83	0	0	1
71	1	0	1
64	0	0	1
54	1	0	2
68	1	0	1
43	1	0	1
83	1	0	1
73	1	1	2
78	0	0	1
80	0	0	2
70	1	0	2
57	1	0	1
75	0	0	2
69	0	1	2
78	1	0	2
69	1	0	1
79	0	0	1
74	1	0	1
91	1	0	2
67	0	0	1
72	1	0	1
67	0	0	1
37	0	0	1
77	1	0	1
69	1	0	2
72	1	0	2
70	1	0	1
75	1	0	1
78	1	0	2
81	1	0	1
47	0	0	1
59	0	0	1
58	1	0	1
75	0	0	1
65	0	0	1
46	0	0	2
59	0	0	2
81	0	0	1
94	0	0	1
85	0	0	1
69	0	0	2
91	1	0	1
70	1	0	1
56	0	0	1
84	1	0	1
84	1	0	1
92	1	0	1
76	0	0	1
69	1	0	1
90	0	0	1
87	1	0	1
78	1	0	1
84	0	0	1
76	0	0	1
81	0	0	1
76	0	0	1
94	0	0	1
78	0	0	1
72	0	0	1
57	1	1	2
76	1	0	2
79	0	1	2
77	1	0	1
60	1	0	1
84	1	0	1
79	1	0	1
70	0	0	1
87	1	0	1
68	0	0	1
75	0	0	1
65	1	0	1
72	0	0	1
50	0	0	2
79	1	0	1
